# The planktonic protist interactome: where do we stand after a century of research?

**DOI:** 10.1038/s41396-019-0542-5

**Published:** 2019-11-04

**Authors:** Marit F. Markussen Bjorbækmo, Andreas Evenstad, Line Lieblein Røsæg, Anders K. Krabberød, Ramiro Logares

**Affiliations:** 1 Department of Biosciences, Section for Genetics and Evolutionary Biology (Evogene), University of Oslo, Blindernv. 31, N-0316 Oslo, Norway; 2 Institut de Ciències del Mar (CSIC), Passeig Marítim de la Barceloneta, 37-49, ES-08003 Barcelona, Catalonia Spain

**Keywords:** Microbial ecology, Theoretical ecology

## Abstract

Microbial interactions are crucial for Earth ecosystem function, but our knowledge about them is limited and has so far mainly existed as scattered records. Here, we have surveyed the literature involving planktonic protist interactions and gathered the information in a manually curated *Protist Interaction DAtabase* (PIDA). In total, we have registered ~2500 ecological interactions from ~500 publications, spanning the last 150 years. All major protistan lineages were involved in interactions as hosts, symbionts (mutualists and commensalists), parasites, predators, and/or prey. Predation was the most common interaction (39% of all records), followed by symbiosis (29%), parasitism (18%), and ‘unresolved interactions’ (14%, where it is uncertain whether the interaction is beneficial or antagonistic). Using bipartite networks, we found that protist predators seem to be ‘multivorous’ while parasite–host and symbiont–host interactions appear to have moderate degrees of specialization. The SAR supergroup (i.e., Stramenopiles, Alveolata, and Rhizaria) heavily dominated PIDA, and comparisons against a global-ocean molecular survey (*TARA Oceans*) indicated that several SAR lineages, which are abundant and diverse in the marine realm, were underrepresented among the recorded interactions. Despite historical biases, our work not only unveils large-scale eco-evolutionary trends in the protist interactome, but it also constitutes an expandable resource to investigate protist interactions and to test hypotheses deriving from omics tools.

## Introduction

Aquatic microbes, including unicellular eukaryotes and prokaryotes, are essential for the functioning of the biosphere [1–4]. Microbes exist in diverse ecological communities where they interact with each other as well as with larger multicellular organisms and viruses.

Interaction between microbial species has played important roles in evolution and speciation. One of the best examples is that the origin of eukaryotes is grounded in the interaction-events of endosymbiosis; giving rise to mitochondria, chloroplasts, and other metabolic capacities in the eukaryotic cell [5–8]. Microbial interactions guarantee ecosystem function, having crucial roles in, for instance, carbon channeling in photosymbiosis, control of microalgae blooms by parasites, and phytoplankton-associated bacteria influencing the growth and health of their host. Despite their importance, our understanding of microbial interactions in the ocean and other aquatic systems is rudimentary, and the majority of them are still unknown [4, 9–11]. The earliest surveys of interactions between aquatic microbes date back to the 19th century. In 1851, while on board H.M.S *Rattlesnake* in the Pacific Ocean, Thomas Huxley discovered small yellow–green cells inside the conspicuous planktonic radiolarians which he thought were organelles [12]. Later on, Karl Brandt established that the yellowish cells were symbiotic alga and named them *Zooxanthella nutricola* [13]. Since these early studies, hundreds of others have reported microbial interactions by using classic tools, mainly microscopy, but this knowledge has not yet been gathered into one accessible database. Over the last ~15 years, HighThroughput Sequencing (HTS) [14–16] of environmental DNA or RNA has transformed our understanding of microbial diversity [17] and evolution [18]. Furthermore, HTS studies have generated hypotheses on microbial interactions based on correlations of estimated microbial abundances over spatiotemporal scales [19–22]. These hypotheses need to be tested with other types of data, such as known interactions from the literature [23]. Overall, HTS will allow to start addressing key driving questions in microbiology such as, what are the main types of interactions in the protist world? Does cooperation outweigh competition among protists? What is the architecture of the protist interactome? And how does this interactome change over spatiotemporal scales?

Here, our main objectives were to assemble the knowledge on aquatic protist interactions from the literature and make it available to the scientific community. We also report the main patterns found in this survey. We examined the available scientific literature spanning the last ~150 years, and recorded ~2500 ecological interactions from ~500 publications going back to the late 1800s [24] (Supplementary Fig. 1). Based on this, we generated a manually curated and publicly available *Protist Interaction DAtabase* (PIDA; 10.5281/zenodo.1195514). PIDA entries have been grouped into four types of pairwise ecological interactions: *parasitism, predation*, *symbiosis*, and ‘*unresolved interaction*’. Parasitism is an antagonistic relationship between organisms, which is beneficial to one partner but harmful to the other, while predation refers for the most part to the engulfment of smaller cells through phagocytosis. In PIDA symbiosis refers to interactions beneficial for both partners (mutualism, e.g., photosymbiosis) or beneficial for one and potentially neutral for the other (commensalism, e.g., host defense). The fourth category ‘unresolved interactions’ are associations where it is uncertain whether the interactions are beneficial or antagonistic to the involved partners. The taxonomic classification in PIDA includes genus and species level, in addition to three levels that were chosen pragmatically to make the database user-friendly and portable.

## Materials and methods

PIDA was assembled between January and November 2017 through a recursive survey of papers on microbial interactions published between 1894 and 2017. The search strategy to find the relevant literature and the template for organizing the database was performed following Lima-Mendez et al. [21]. Initially, reviews resulting from the Boolean search string (plankton* AND (marin* OR ocean*)) AND (parasit* OR symbios* OR mutualis*) in Scopus (https://www.scopus.com/) and Web of Science (http://webofknowledge.com/) were examined, then the references therein were further explored. In addition, literature on protist predation on other protists and bacteria were also screened. Entries from the *AquaSymbio* database (http://aquasymbio.fr/) were compared against the entries in PIDA, and occasionally used as a source of additional literature. The overlap between *AquaSymbio* and PIDA is ~20% (~500 entries). Many of the entries in *AquaSymbio* are interactions between protists and multicellular organisms, therefore they are not included in PIDA.

PIDA documents the ecological interaction between two organisms, identified down to the species level, if possible. Interactions are characterized as *parasitism*, *predation*, *symbiosis* (either mutualism or commensalism), or ‘*unresolved*’. Parasitism is used in cases where the study clearly identifies a parasitic interaction. Cases of kleptoplasty and mixotrophy together with classical predation are contained within the group of entries termed predation. Symbiosis includes endo- and ectosymbiosis and is categorized into the different forms of symbiosis (e.g., photosymbiosis). The unresolved interactions include associations/interactions between organisms where it is yet unknown whether the associations are beneficial or antagonistic.

In addition to genus and species levels, the taxonomic classification includes three additional levels chosen pragmatically to make the database more user-friendly and portable. The highest level distinguishes between eukaryotes and prokaryotes. The second level places each taxon within supergroups or other high taxonomic ranks (e.g., Rhizaria or Alveolata) following the scheme of Adl et al. [25, 26]. The third level places each taxon in groups below the supergroup taxonomic rank (phylum, e.g., Ciliophora, Dinoflagellata, and Acantharia, or class levels, e.g., Chlorophyceae, Kinetoplastea, and Diplomonadida). The taxonomic names at the third level follows the nomenclature of the SILVA database (release 128, May/June 2017) [27–29]. Species names in PIDA have been updated to the most recent agreed-upon classification and can therefore deviate from the original papers they stem from due to synonymization. PIDA also documents the methods used to determine the interacting species. Symbionts and/or hosts determined by any form of microscopy or direct observation are denoted (1). Symbionts and/or hosts determined by sequencing or Fluorescence In Situ Hybridization (FISH) are denoted (2). The combination of the former two is denoted (3). Most interactions with observation type 2 also have GenBank [30] accession numbers. A published paper is associated to each interaction entry, and when a DOI is available, it is included. Only interactions from aquatic systems are included (marine, brackish, and freshwater). The resulting PIDA contains 2422 entries from 528 publications and is publicly available at github (https://github.com/ramalok/PIDA).

### Bipartite networks

Bipartite networks are the representation of interactions between two distinct classes of nodes, such as plant–pollinator, parasite–host, or prey–predator. Identifying patterns in bipartite networks is useful in explaining their formation and function. We investigated how symbiosis, parasitism, and predation differ in terms of specialization. For example, if parasite taxa have a broader host range compared with the host range of symbionts, this indicates that parasites are less specialized (and consequently more generalists) than symbionts. We also used the bipartite networks to investigate whether predators are omnivorous (generalists) or picky (specialists) in their diets. All analyses were conducted in the statistical environment R v. 3.5.0 [31]. We constructed bipartite qualitative (binary) directional networks using the R-package *bipartite* v. 2.08 [32]. All taxa where the taxonomy assigned to one of the ‘partners’ in PIDA was ‘unknown eukaryote’, ‘unidentified bacteria’, or ‘unidentified prokaryote’ were removed before further analyses of the bipartite networks. Bipartite network indices were calculated using the functions *networklevel* [33] and *specieslevel* [34] (default settings except weighted = FALSE) in the R-package *bipartite*. Bipartite networks and network analyses were performed at four taxonomic levels (‘supergroup’, ‘phylum’, genus, and species). Found patterns were consistent across the taxonomic levels, therefore only the species level is shown. Degree (number of links/edges/interactions per node) was calculated for prey, predators, parasites, symbionts, ‘interactors’, and hosts at the species level. The specialization index *d*’ (Kullback–Leibler distance) [35], measures the degree of specialization at the species level, and was calculated as deviation of the actual interaction frequencies from a null model that assumes all partners in the other level of the bipartite network are used in proportion to their availability. The specialization index *d*’ ranges from 0 for the most generalist to 1 for the most specialist, and was calculated for prey, predators, parasites, symbionts, ‘interactors’, and hosts at the species level.

**Fig. 1:**
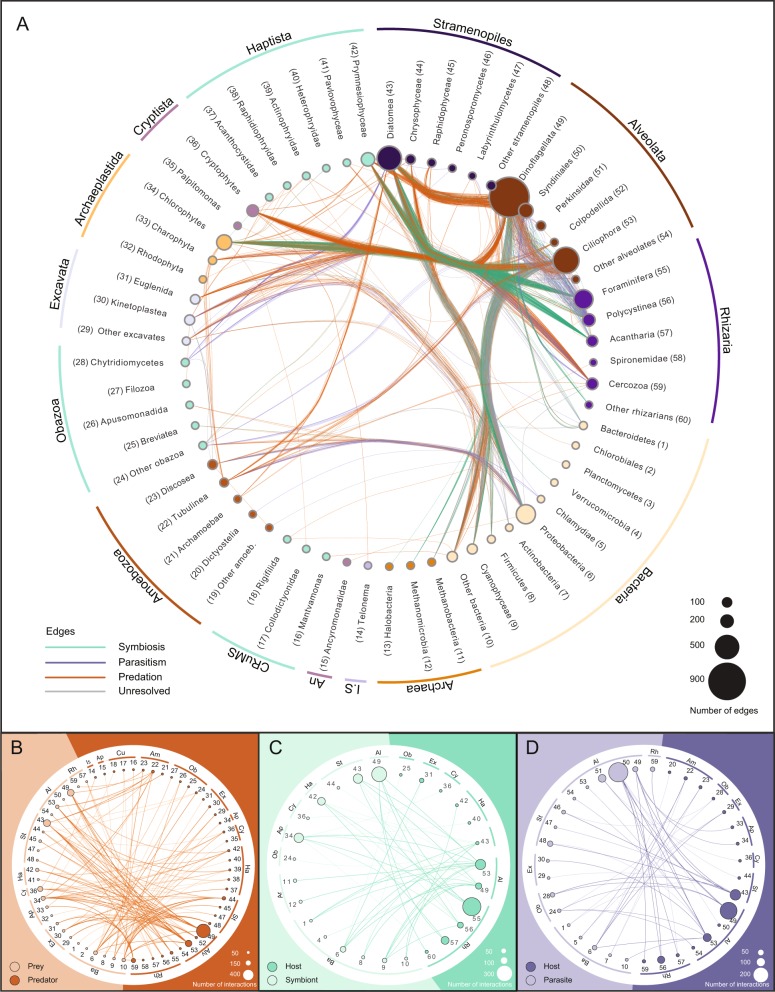
Overview of the interactions included in PIDA. Nomenclature and taxonomic order of Eukaryota is based on Adl et al. 2019 [25]. Nomenclature and taxonomic order of Bacteria is based on Schultz et al. 2017 [87]. The nodes are grouped (outer circle) according to eukaryotic supergroups (or *Incertae sedis*), Bacteria and Archaea. **a** Network based on the 2422 entries in PIDA. Nodes represent eukaryotic and prokaryotic taxa and are colored accordingly. Node size indicates the number of edges/links that are connected to that node. Each node/taxon is assigned a number, which corresponds with the numbers for taxa in **b–d**. Edges represent interactions between two taxa and are colored according to ecological interaction type: *predation* (orange), *symbiosis* (green), and *parasitism* (purple). The network is undirected, meaning that a node can contain both parasites/symbionts/prey and hosts/predators. To avoid cluttering of the figure, ‘Self-loops’, which represent cases where both interacting organisms belong to the same taxon (e.g., a dinoflagellate eating another dinoflagellate) are not shown as edges/links in this figure, but are considered in the size of nodes. The outermost circle groups taxa in the different eukaryotic ‘supergroups’ or the prokaryotic domains Bacteria and Archaea. Ancryomonadidae is abbreviated An. Telonema is not placed into any of the supergroups, but classified as *Incertae sedis* (abbreviated *I*.*S*. in the figure). In **b**, **c**, and **d** the following abbreviations for supergroups are used: Ar Archaea, Ba Bacteria, Rh Rhizaria, Al Alveolata, St Stramenopiles, Ha Haptista, Cy Cryptista, Ap Archaeplastida, Ex Excavata, Ob Obazoa, Am Amoebozoa, Cu CRuMS, An Ancryomonadidae, Is *Incertae sedis*. **b** Predator–prey interactions in PIDA. The node numbers correspond to taxa node numbers in **a**. Abbreviations for supergroups are described above. Background and nodes are colored according to functional role in the interaction: Prey are colored light orange (left part of figure), while predators are depicted in dark orange (right part of figure). The size of each node represents the number of edges connected to that node. **c** Symbiont–host interactions included in PIDA. The node numbers correspond to node numbers in **a**. Abbreviations for supergroups are described above. Symbionts are to the left, colored light green, and their hosts are to the right in dark green. The size of each node represents the number of edges connected to that node. **d** Parasite–host interactions included in PIDA. The node numbers correspond to node numbers in **a**. Abbreviations for supergroups are described above. Parasite taxa are depicted in light purple (left), hosts in dark purple (right). (The unresolved interactions are shown in Supplementary Fig. 7)

All barplots and density plots were constructed using the R-package *ggplot2* v. 3.1.0 [36] and the networks in Fig. 1a–d and Supplementary Fig. 2 were visualized in CytoScape v. 3.6.1 [32, 37].

### Interlinked species

Interlinked species are taxa that are present in either several types of interactions, or on both sides of the same interaction. Interlinked species were determined using the R-package *systemPipeR* v. 3.8 [38] for all interaction types, except the unresolved interactions (since the nature of these interaction is unknown). Only taxa with full species names were included to avoid overestimating overlapping species (e.g., *Amoebophrya* sp. and similar were excluded from the list of overlapping species). Venn diagram intersects were computed using the function *overLapper* and plotted using the function *vennPlot*. Parasites only overlapped with parasite hosts and were subsequently added to the Venn plot.

## Results

### Aquatic microbial interactions

The literature in PIDA was dominated by studies based on direct observation of interactions such as light microscopy. In total, 82% of the entries were based on microscopy, and only 38% of those were combined with molecular methods. The most commonly studied interaction in the literature was predation, representing 39% of all entries, followed by symbiosis (29%), parasitism (18%), and unresolved interactions (14%).

**Fig. 2:**
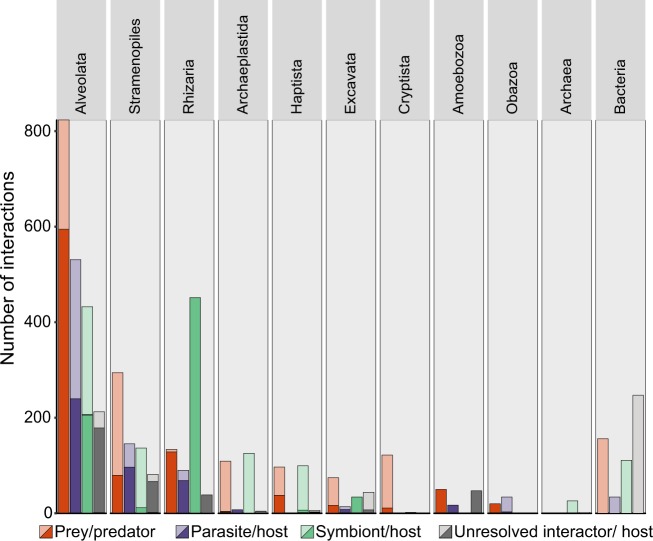
Interactions in PIDA. Number of interactions registered in the PIDA database for the different taxonomic groups at ‘supergroup level’ (corresponding to the second taxonomic level in PIDA). Red bars show predation, purple parasitism, green represent symbiosis, and gray unresolved interactions. Solid colors represent predator/host and transparent colors represent prey/symbiont/parasite/interactor. Because CRuMS, Ancyromonadidae, and *Incertae sedis* comprised very few entries (5, 1, and 2 predator entries, respectively), they are not included in this figure

The SAR supergroup (Alveolata, Stramenopiles, and Rhizaria) dominated with ~92% of the total entries (Figs. 1 and 2). Of all host and predator records, ~90% belonged to the SAR supergroup (Alveolata 51%, Stramenopiles 12%, and Rhizaria 27%; Fig. 2). The SAR supergroup was less dominant as symbiont/ parasite/ interactor/ prey, but still represented the largest group, with 50% of all entries (Alveolata 33%, Stramenopiles 16%, and Rhizaria 1%; Fig. 2).

The majority of interactions (82%) were from marine or brackish waters, while studies from freshwater systems accounted for a smaller fraction of the interactions (18%). This is not surprising given the larger number of studies from the marine phototrophic zone compared to other environments.

### Predator–prey interactions

Predator–prey interactions constituted the majority of entries in PIDA. We acknowledge that separating predation into categories represents a simplified version of how predator–prey interactions are in nature, where e.g., mixotrophy is challenging old paradigms [39–42]. However, for simplicity, we here divided predation into three different categories depending on the type of prey involved: herbivory (grazing on autotrophic/chloroplast containing eukaryotic algae), bacterivory (feeding on autotrophic and/or heterotrophic bacteria) or ‘carnivory’ (predation on other heterotrophic protists). Based on this definition, herbivory was the most common form of predation in our survey (68% of the predator–prey interactions) with all the major eukaryotic lineages represented among the predators. Entries of herbivore dinoflagellates and ciliates (both Alveolata) dominated (Fig. 1a, b). Bacterivory accounted for 16% of the predator–prey interactions and was also documented in most eukaryotic groups (Fig. 1b), with an expected predominance of small heterotrophic flagellates.

### Symbiont–host interactions

Symbiotic *protist–protist* interactions made up 81% of the symbiont entries and all of these interactions represented photosymbiosis. Dinoflagellates, diatoms, chlorophyceans, trebouxiophyceans, and prymnesiophytes accounted for most of the recorded photosymbionts, living in symbiosis with rhizarian, ciliate, and dinoflagellate hosts (Fig. 1a, c). *Bacteria–protist* interactions represented 16% of the total number of symbiont entries in PIDA, and was dominated by bacterial entries belonging to Proteobacteria and Cyanophyceae that mainly interacted with Alveolata (dinoflagellates and ciliates), Stramenopiles (diatoms), and Excavata (euglenids); Fig. 1a, c. The bacteria–protist interactions were involved in many different types of symbiotic relationships, from photosymbiosis (13%) to nitrogen fixation (46%) and vitamin exchange (36%). Symbiotic *archaea–protist* interactions represented 3% of symbiont entries in PIDA, and the majority of these were methanogenic symbiont interactions between archaeal Metanomicrobia and anaerobic Ciliophora (Fig. 1a, c).

### Unresolved interactions

The unresolved interactions represent all ecological interactions where the functional role of the relationship between the partners was not determined. Several of these cases likely represent commensalism. The unresolved ‘interactor’–host category mainly consisted of *protist-bacteria* interactions (73%), dominated by interactions between Proteobacteria and Alveolata (ciliates and dinoflagellates) or Proteobacteria and Stramenopiles (diatoms; Fig. 1a & Supplementary Fig. 2). *Protist–protist* interactions represented 27% of the unresolved interactions, and mainly included alveolate, excavate, and stramenopile symbionts that interacted with alveolate, rhizarian, stramenopile, and amoebozoan hosts (Fig. 1a & Supplementary Fig. 2). The amoebozoan hosts (*Neoparamoeba* spp.) were only registered to interact with unknown kinetoplastids (Excavata), which is likely an example of an unusual form of endosymbiosis [43]. The biological nature of these interactions still remains unknown.

### Parasite–host interactions

Parasites in PIDA were dominated by a few taxonomic groups that all belonged to Alveolata, such as Syndiniales (~50% *Amoebophrya*), Perkinsidae (~98% *Parvilucifera*), and Dinoflagellata. Together they accounted for 2/3 of the parasite entries (Fig. 1a, d). These alveolate parasites mainly infected other alveolates such as dinoflagellates and ciliates, but rhizarian and diatom hosts were also recorded (Fig. 1d). Parasites belonging to different stramenopiles lineages such as Peronosporomycetes (oomycetes), Labyrinthulomycetes, and *Pirsonia* were mainly described from diatom hosts (Fig. 1d). Rhizarian parasites constituted 5% of the parasite records and were represented by just a few cercozoans and phagomyxids, which parasitized diatoms, as well as the rhizarian phytomyxid *Woronina pythii*, which parasitized different *Pythium* species (Perenosporomycetes). Parasitic fungi from Chytridiomycetes, Microsporidia, and Sordariomycetes (the last two included in ‘other Obazoa’ in Fig. 1a, d) were also represented by relatively few entries (only 7% of the parasite records). Yet, the records of parasitic fungi demonstrated that they infect a relatively broad range of protists, such as dinoflagellates, apicomplexans, ciliates, and diatoms. Bacterial parasites of protists accounted for 8% of the parasite entries and were registered mainly from amoebozoan, excavate, and ciliate hosts (Fig. 1d).

### Bipartite interaction networks

**Fig. 3:**
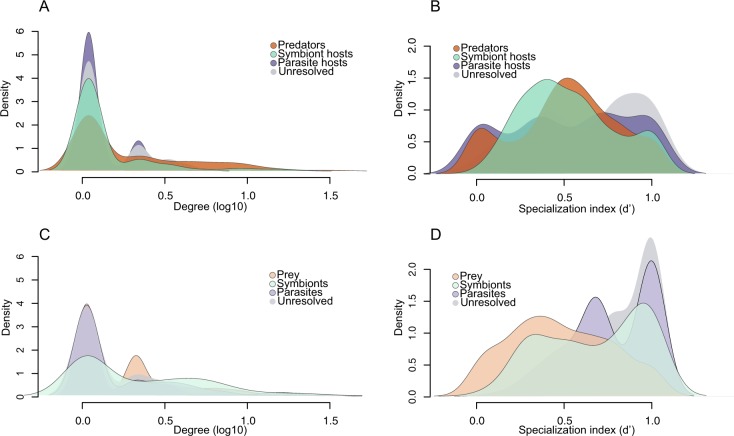
Density plots for degree and specialization indices for the bipartite networks in PIDA. **a** Degree (number of links/edges/interactions) for predators, hosts of parasites, hosts of symbionts, and hosts of interactors in the bipartite networks. **b** Specialization index *d*’ (Kullback–Leibler distance) [35], for predators, hosts of parasites, hosts of symbionts, and host of interactors. The specialization index *d*’ ranges from 0 for the most generalized to 1 for the most specialized. **c** Degree (number of links/edges/interactions) for prey, parasites, symbionts, and interactors. **d** Specialization index *d*’ for prey, parasites, symbionts, and interactors

Since PIDA consists of pairwise interactions between aquatic microbes where the roles of the participants are known we can represent the interactions as bipartite networks. Bipartite networks provide a systematic way of representing data that consist of two distinct guilds, such as plant–pollinator, parasite–host, or predator–prey. These networks are composed of nodes (representing species or genera) connected by links (edges) representing the interactions between nodes. The *degree* of a node (species) is the sum of links connecting the particular node to the nodes from the other guild. Consequently, a higher degree value indicates a higher level of generalism [33]. For example, a parasite that has gone through multiple host-shifts and has the capacity to parasitize different hosts would display a higher degree than a parasite specialized to interact with only one host. We have constructed binary (presence/absence) bipartite networks for predator–prey, symbiont–host, and parasite–host interactions, as well as for the unresolved interactions (Supplementary Figs. 3–6). We calculated specialization indices to analyze variation in specialization within the bipartite networks and to examine if the four interaction types differed in terms of specialization (Fig. 3a–d; Supplementary Fig. 7; Table 1).

**Table 1:** Degree and specialization index (*d*’), for the five species with highest degree (highest number of links/edges) in the bipartite networks for each interaction type

Type	Taxa^a^	Species	Phylum (Supergroup)	Degree	*d*'
Predators	337	*Leptophrys vorax*	Cercozoa (Rhizaria)	34	0.8
		*Oblea rotunda*	Dinoflagellata (Alveolata)	27	0.6
		*Karlodinium armiger*	Dinoflagellata (Alveolata)	26	0.6
		*Diplopsalis lenticula*	Dinoflagellata (Alveolata)	15	0.4
		*Diplopsalopsis bomba*	Dinoflagellata (Alveolata)	15	0.4
Hosts of symbionts	188	*Amphistegina lobifera*	Foraminifera (Rhizaria)	17	0.6
		*Amphistegina lessonii*	Foraminifera (Rhizaria)	15	0.6
		*Borelis schlumbergi*	Foraminifera (Rhizaria)	12	0.6
		*Heterostegina depressa*	Foraminifera (Rhizaria)	12	0.5
		*Paramecium bursaria*	Ciliophora (Alveolata)	9	0.8
Hosts of interactors	141	*Diophrys scutum*	Ciliophora (Alveolata)	4	0.2
		*Euplotes harpa*	Ciliophora (Alveolata)	4	0.9
		*Euplotes woodruffi*	Ciliophora (Alveolata)	4	0.7
		*Hemigastrostyla elongata*	Ciliophora (Alveolata)	4	0.3
		*Petalomonas spagnophila*	Euglenida (Excavata)	4	0.4
Hosts of parasites	262	*Alexandrium minutum*	Dinoflagellata (Alveolata)	5	0.3
		*Collozoum* NA	Polycystinea (Rhizaria)	5	0.8
		*Guinardia delicatula*	Diatomea (Stramenopiles)	5	0.6
		*Alexandrium catenella*	Dinoflagellata (Alveolata)	4	0.1
		*Coscinodiscus granii*	Diatomea (Stramenopiles)	4	0.5
Prey	342	*Isochrysis galbana*	Prymnesiophyceae (Haptista)	31	0.3
		*Rhodomonas salina*	Cryptophyceae (Cryptista)	27	0.4
		*Skeletonema costatum*	Diatomea (Stramenopiles)	27	0.3
		*Heterocapsa triquetra*	Dinoflagellata (Alveolata)	20	0.3
		*Heterosigma akashiwo*	Raphidiophyceae (Stramenopiles)	19	0.2
Symbionts	98	*Phaeocystis* NA	Prymnesiophyceae (Haptista)	29	1.0
		diatom NA	Diatomea (Stramenopiles)	21	0.9
		cyanophyte NA	Cyanophyceae (Cyanobacteria)	18	0.9
		dinoflagellate NA	Dinoflagellata (Alveolata)	15	0.9
		Chlorella NA	Trebouxiophyceae (Chlorophyta)	11	0.7
Interactors	85	alphaproteobacter NA	Alphaproteobacteria (Proteobacteria)	20	0.5
		gammaproteobacteria NA	Gammaproteobacteria (Proteobacteria)	19	0.6
		*Gyrodinium NA*	Dinoflagellata (Alveolata)	12	1.0
		cyanophyte NA	Cyanophyceae (Cyanobacteria)	8	1.0
		*Polynucleobacter necessarius*	Betaproteobacteria (Proteobacteria)	7	0.8
Parasites	130	*Amoebophrya* NA	Syndiniales (Alveolata)	61	0.8
		*Parvilucifera infectans*	Perkinsidae (Alveolata)	29	0.5
		*Euduboscquella* NA	Syndiniales (Alveolata)	18	1.0
		*Cryothecomonas longipes*	Cercozoa (Rhizaria)	14	0.6
		*Pirsonia formosa*	*I*.*s*. Stramenopiles	14	0.6

^a^Taxa displays the number of species registered in PIDA for the different interaction types. The taxonomy of the five species with highest degree is shown at species, phylum, and supergroup level. Degree shows the number of edges for the top five taxa. Specialization index *d*’ (Kullback–Leibler distance) [30] ranges from 0 for the most generalized to 1 for the most specialized. Abbreviations used: *Type* interaction type.

The *predator–prey* bipartite interaction networks had 342 prey and 337 predator species (Supplementary Fig. 3). Although the number of prey and predators in the network were almost equal, there were multiple shared interactions. That is, several predators feed on the same prey (i.e., the prey has a high degree) and conversely, generalist predators preying on multiple prey organisms (i.e., predators having a high degree; Fig. 3a, c; Supplementary Figs. 3 and 7). The five predators with highest degree included four dinoflagellates and one cercozoan (Table 1).

The prey organisms with highest degree belonged to Haptista, Cryptista, Stramenopiles, and Alveolata (Table 1). The specialization index (*d’*) was uniformly distributed from 0 (generalist) to 1 (specialist) indicating that predation was not driving predator or prey to specialization (Fig. 3b, d).

The *symbiont–host* interaction networks consisted of 98 symbiont and 188 host species (Supplementary Fig. 4). The majority of both hosts and symbionts had a low degree (Fig. 3a, c; Supplementary Fig. 7). The distribution of the specialization index (*d*’) for hosts indicates that PIDA includes both, specialists interacting with only one or a few symbionts, as well as hosts that interact with multiple symbionts (Fig. 3b). The five host taxa that had the highest number of associated symbionts (i.e., highest degree) were four foraminiferans (Rhizaria) and one ciliate (Alveolata; Table 1). Very few hosts were ‘true generalists’ (i.e., with *d*’ close to 0, Fig. 3b). The symbionts in PIDA had high *d*’ values in general, which indicate that they are specialized (*d*’ 0.75–1; Fig. 3d). This was coherent with the low degree of most symbionts, which showed few links to different hosts (Fig. 3c; Supplementary Fig. 7). The five symbionts with highest degree included taxa belonging to five different ‘supergroups’ (Haptista, Stramenopiles, Cyanobacteria, Alveolata, and Chlorophyta; Table 1).

The network for the *unresolved interactor–host* interactions had 85 ‘interactors’ and 141 host species (Supplementary Fig. 5). The distribution of the specialization index (*d*’) for hosts and interactors showed high *d*’ values for most taxa (Fig. 3b, d). Concordantly, the majority of hosts and interactors also had a low degree (Fig. 3a, c; Supplementary Fig. 7). Altogether, this could indicate that the majority of the *unresolved interactor–host* interactions are specialized, or that they are simply understudied. The five interactors and the five hosts with highest degree are shown in Table 1.

The network for *parasitism* had 130 parasites and 262 hosts (Supplementary Fig. 6). Hosts were dominated by taxa with low degree (i.e., few parasites per host, maximum number was five; Table 1), which indicated that they are infected by a relatively low number of parasites (Fig. 3a; Supplementary Fig. 7). The *d*’ values showed, however, that there was an equal distribution of host taxa ranging from ‘true generalists’ (*d*’ value of 0) to ‘true specialists’ (*d*’ value of 1; Fig. 3b). The parasites had for the most part a low degree, and the distribution of the specialization index indicated that several of the parasites were specialists (*d*’ values ~1; Fig. 3d). Parasites showed the highest relative number of specialized taxa in PIDA. However, parasites also included the taxa with the highest degree (Fig. 3c; Supplementary Fig. 7), the well-known parasites belonging to Syndiniales (MALV II) and Perkinsidae (Table 1).

### Interlinked species

**Fig. 4:**
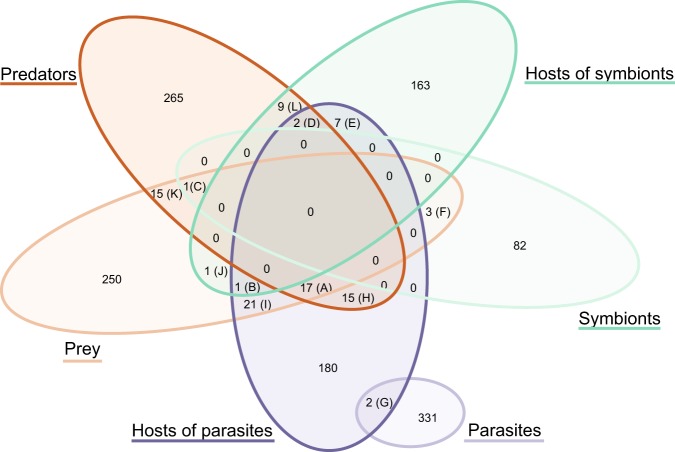
Interlinked species. Venn diagram illustrating the number of species in PIDA that hold multiple ‘roles’ and are present in more than one interaction network/type; i.e., either present in two distinct networks (e.g., registered as ‘Prey’ in the predator–prey network, and also as ‘Symbiont’ in the symbiont–host network) or on both sides of the same network (e.g., registered as both ‘Predator’ and ‘Prey’ in the predator–prey network). Letters A–L refers to the panels in Table 2, where the taxonomy of the different overlapping species and their roles are presented. ‘Parasites’ were only found to overlap with ‘Host of parasites’, representing two cases of hyperparasitism (i.e., parasite species parasitizing other parasite species). The unresolved interactions are not included in this analysis since their functional roles are unknown. Only taxa with full species name determined were included to avoid overestimating overlapping species (e.g., *Amoebophrya* NA and similar were excluded)

Interlinked species [44] are taxa present in either several types of interactions, or on both sides of the same interaction. An interlinked species is for example a species that is registered as a predator in the predator–prey network, and is also present as a host in the symbiont–host network. The unresolved interactions were excluded from these analyses since the nature of these interactions are unknown. In total there were 94 interlinked species in PIDA (~4% of the total entries, Fig. 4; Table 2). The maximum number of interaction types for any species was three (Table 2; panels A–D). The majority of interlinked species occurred in the overlap of species recorded as predator, as prey and as host of parasites (Table 2; panel A). There was only a single interlinked species that held a role in each of the three independent bipartite networks (i.e., predator–prey, symbiont–host, and parasite–host), *Paramecium bursaria* (Table 2, panel B; corresponding to the overlap B in Fig. 4). The majority of interlinked species had two interaction types (Table 2; panels E–L). There were also two examples of hyperparasitism where the species were parasites as well as hosts of other parasites (Table 2; panel G).

**Table 2:** Interlinked species in PIDA

Panel	Species	Phylum	Supergroup	No	Present as		
A	*Akashiwo sanguinea*	Dinoflagellata	Alveolata	3	Prey	Predator	Host of par
A	*Alexandrium catenella*	Dinoflagellata	Alveolata	3	Prey	Predator	Host of par
A	*Alexandrium ostenfeldii*	Dinoflagellata	Alveolata	3	Prey	Predator	Host of par
A	*Alexandrium tamarense*	Dinoflagellata	Alveolata	3	Prey	Predator	Host of par
A	*Cochlodinium polykrikoides*	Dinoflagellata	Alveolata	3	Prey	Predator	Host of par
A	*Dinophysis acuminata*	Dinoflagellata	Alveolata	3	Prey	Predator	Host of par
A	*Gymnodinium catenatum*	Dinoflagellata	Alveolata	3	Prey	Predator	Host of par
A	*Gymnodinium sanguineum*	Dinoflagellata	Alveolata	3	Prey	Predator	Host of par
A	*Heterocapsa rotundata*	Dinoflagellata	Alveolata	3	Prey	Predator	Host of par
A	*Heterocapsa triquetra*	Dinoflagellata	Alveolata	3	Prey	Predator	Host of par
A	*Neoceratium furca*	Dinoflagellata	Alveolata	3	Prey	Predator	Host of par
A	*Oblea rotunda*	Dinoflagellata	Alveolata	3	Prey	Predator	Host of par
A	*Oxyrrhis marina*	Dinoflagellata	Alveolata	3	Prey	Predator	Host of par
A	*Prorocentrum micans*	Dinoflagellata	Alveolata	3	Prey	Predator	Host of par
A	*Prorocentrum minimum*	Dinoflagellata	Alveolata	3	Prey	Predator	Host of par
A	*Protoperidinium pellucidum*	Dinoflagellata	Alveolata	3	Prey	Predator	Host of par
A	*Scrippsiella trochoidea*	Dinoflagellata	Alveolata	3	Prey	Predator	Host of par
B	*Paramecium bursaria*	Ciliophora	Alveolata	3	Prey	Host of symb	Host of par
C	*Heterocapsa rotunda*	Dinoflagellata	Alveolata	3	Symbiont	Prey	Predator
D	*Noctiluca scintillans*	Dinoflagellata	Alveolata	3	Predator	Host of symb	Host of par
D	*Thalassicolla nucleata*	Polycystinea	Rhizaria	3	Predator	Host of symb	Host of par
E	*Durinskia baltica*	Dinoflagellata	Alveolata	2	Host of symb	Host of par	–
E	*Durinskia dybowskii*	Dinoflagellata	Alveolata	2	Host of symb	Host of par	–
E	*Kryptoperidinium foliaceum*	Dinoflagellata	Alveolata	2	Host of symb	Host of par	–
E	*Stentor polymorphus*	Ciliophora	Alveolata	2	Host of symb	Host of par	–
E	*Acanthometra pellucida*	Acantharia	Rhizaria	2	Host of symb	Host of par	–
E	*Acanthostaurus purpurascens*	Acantharia	Rhizaria	2	Host of symb	Host of par	–
E	*Collozoum caudatum*	Polycystinea	Rhizaria	2	Host of symb	Host of par	–
F	*Chlamydomonas hedleyi*	Chlorophyceae	Archaeplastida	2	Prey	Symbiont	–
F	*Nitzschia frustulum*	Diatomea	Stramenopiles	2	Prey	Symbiont	–
F	*Protodinium simplex*	Dinoflagellata	Alveolata	2	Prey	Symbiont	–
G	*Keppenodinium mycetoides*	Syndiniales	Alveolata	2	Parasite	Host of par	–
G	*Oodinium acanthometrae*	Dinoflagellata	Alveolata	2	Parasite	Host of par	–
H	*Acanthamoeba castellanii*	Discosea	Amoebozoa	2	Predator	Host of par	–
H	*Acanthamoeba polyphaga*	Discosea	Amoebozoa	2	Predator	Host of par	–
H	*Dinophysis acuta*	Dinoflagellata	Alveolata	2	Predator	Host of par	–
H	*Dinophysis norvegica*	Dinoflagellata	Alveolata	2	Predator	Host of par	–
H	*Diplopsalis lenticula*	Dinoflagellata	Alveolata	2	Predator	Host of par	–
H	*Euplotes woodruffi*	Ciliophora	Alveolata	2	Predator	Host of par	–
H	*Eutintinnus pectinis*	Ciliophora	Alveolata	2	Predator	Host of par	–
H	*Favella ehrenbergii*	Ciliophora	Alveolata	2	Predator	Host of par	–
H	*Gonyaulax polygramma*	Dinoflagellata	Alveolata	2	Predator	Host of par	–
H	*Karlodinium veneficum*	Dinoflagellata	Alveolata	2	Predator	Host of par	–
H	*Trithigmostoma cucullulus*	Ciliophora	Alveolata	2	Predator	Host of par	–
H	*Protoperidinium bipes*	Dinoflagellata	Alveolata	2	Predator	Host of par	–
H	*Protoperidinium minutum*	Dinoflagellata	Alveolata	2	Predator	Host of par	–
H	*Protoperidinium steinii*	Dinoflagellata	Alveolata	2	Predator	Host of par	–
H	*Strombidium capitatum*	Ciliophora	Alveolata	2	Predator	Host of par	–
I	*Coscinodiscus granii*	Diatomea	Stramenopiles	2	Prey	Host of par	–
I	*Coscinodiscus radiatus*	Diatomea	Stramenopiles	2	Prey	Host of par	–
I	*Cylindrotheca closterium*	Diatomea	Stramenopiles	2	Prey	Host of par	–
I	*Eucampia zoodiacus*	Diatomea	Stramenopiles	2	Prey	Host of par	–
I	*Chaetoceros didymus*	Diatomea	Stramenopiles	2	Prey	Host of par	–
I	*Guinardia delicatula*	Diatomea	Stramenopiles	2	Prey	Host of par	–
I	*Guinardia flaccida*	Diatomea	Stramenopiles	2	Prey	Host of par	–
I	*Leptocylindrus danicus*	Diatomea	Stramenopiles	2	Prey	Host of par	–
I	*Stephanopyxis turris*	Diatomea	Stramenopiles	2	Prey	Host of par	–
I	*Thalassionema nitzschioides*	Diatomea	Stramenopiles	2	Prey	Host of par	–
I	*Thalassiosira nordenskioeldii*	Diatomea	Stramenopiles	2	Prey	Host of par	–
I	*Thalassiosira punctigera*	Diatomea	Stramenopiles	2	Prey	Host of par	–
I	*Thalassiosira rotula*	Diatomea	Stramenopiles	2	Prey	Host of par	–
I	*Odontella sinensis*	Diatomea	Stramenopiles	2	Prey	Host of par	–
I	*Gymnodinium instriatum*	Dinoflagellata	Alveolata	2	Prey	Host of par	–
I	*Gymnodinium mikimotoi*	Dinoflagellata	Alveolata	2	Prey	Host of par	–
I	*Gyrodinium aureolum*	Dinoflagellata	Alveolata	2	Prey	Host of par	–
I	*Neoceratium fusus*	Dinoflagellata	Alveolata	2	Prey	Host of par	–
I	*Neoceratium lineatum*	Dinoflagellata	Alveolata	2	Prey	Host of par	–
I	*Neoceratium tripos*	Dinoflagellata	Alveolata	2	Prey	Host of par	–
I	*Paramecium tetraurelia*	Ciliophora	Alveolata	2	Prey	Host of par	–
J	*Thalassiosira pseudonana*	Diatomea	Stramenopiles	2	Prey	Host of symb	–
K	*Heterosigma akashiwo*	Raphidophyceae	Stramenopiles	2	Prey	Predator	–
K	*Pseudobodo tremulans*	Bicosoecida	Stramenopiles	2	Prey	Predator	–
K	*Fibrocapsa japonica*	Raphidophyceae	Stramenopiles	2	Prey	Predator	–
K	*Archerella flavum*	Labyrinthulomycetes	Stramenopiles	2	Prey	Predator	–
K	*Lingulodinium polyedrum*	Dinoflagellata	Alveolata	2	Prey	Predator	–
K	*Myrionecta rubra*	Ciliophora	Alveolata	2	Prey	Predator	–
K	*Pfiesteria piscicida*	Dinoflagellata	Alveolata	2	Prey	Predator	–
K	*Prorocentrum triestinum*	Dinoflagellata	Alveolata	2	Prey	Predator	–
K	*Euplotes aediculatus*	Ciliophora	Alveolata	2	Prey	Predator	–
K	*Favella taraikaensis*	Ciliophora	Alveolata	2	Prey	Predator	–
K	*Amphidinium carterae*	Dinoflagellata	Alveolata	2	Prey	Predator	–
K	*Rhyncomonas nasuta*	Kinetoplastea	Excavata	2	Prey	Predator	–
K	*Bodo saltans*	Kinetoplastea	Excavata	2	Prey	Predator	–
K	*Prymnesium parvum*	Prymnesiophyceae	Haptophyta	2	Prey	Predator	–
K	*Chrysochromulina polylepis*	Prymnesiophyceae	Haptophyta	2	Prey	Predator	–
L	*Peridinium quinquecorne*	Dinoflagellata	Alveolata	2	Predator	Host of symb	–
L	*Euplotes daidaleos*	Ciliophora	Alveolata	2	Predator	Host of symb	–
L	*Climacostomum virens*	Ciliophora	Alveolata	2	Predator	Host of symb	–
L	*Orbulina universa*	Foraminifera	Rhizaria	2	Predator	Host of symb	–
L	*Archaias angulatus*	Foraminifera	Rhizaria	2	Predator	Host of symb	–
L	*Globigerinoides ruber*	Foraminifera	Rhizaria	2	Predator	Host of symb	–
L	*Globigerinoides sacculifer*	Foraminifera	Rhizaria	2	Predator	Host of symb	–
L	*Globorotalia menardii*	Foraminifera	Rhizaria	2	Predator	Host of symb	–
L	*Lenisia limosa*	Breviatea	Obazoa	2	Predator	Host of symb	–

**Fig. 5:**
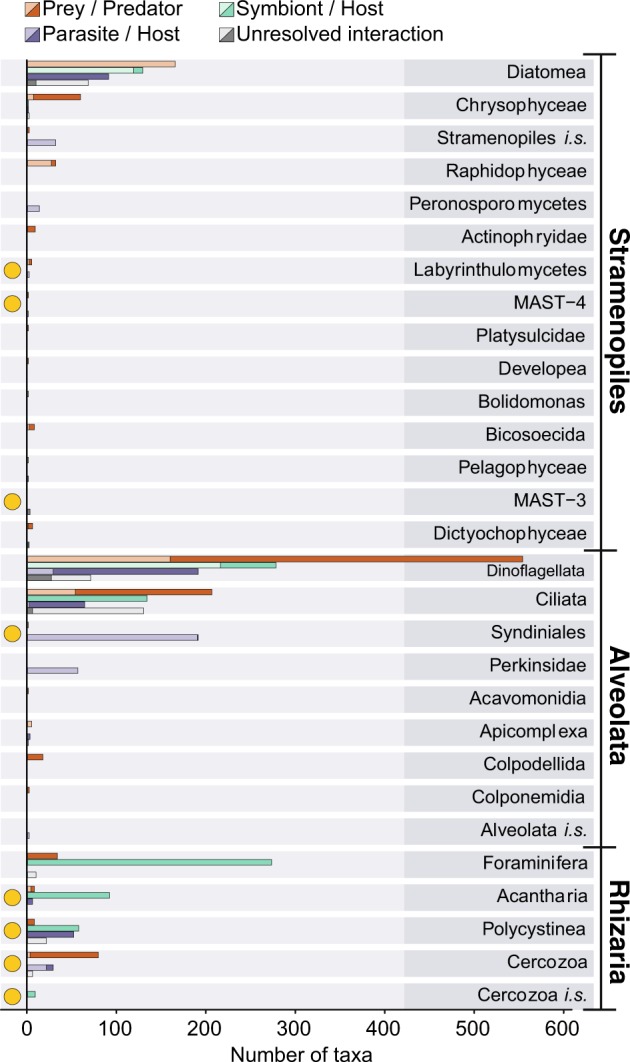
The dominating SAR supergroup. Number of interactions registered in PIDA belonging to the SAR supergroup (Stramenopiles, Alveolates, and Rhizaria). For each of the SAR supergroups the entries of the different taxonomic groups at ‘phylum level’ are shown (corresponding to the third taxonomic level in PIDA). Red bars show predators, green represent symbiosis, purple parasitism, and gray unresolved interactions. Solid colors represent host/predator and transparent colors represent prey/symbiont/parasite/interactor. Yellow circles highlight the ‘phyla’ that comprise few records in PIDA compared with the (hyper)diversity these ‘phyla’ represent in environmental HTS studies, such as the *Tara Oceans* study [45]. Abbreviations used in this figure: *i*.*s*. refers to *Incertae sedis* or unknown

Panels A–L refer to the 12 overlapping sections in the Venn diagram (Fig. 5). The taxonomy of the overlapping species in PIDA is listed at species, phylum and supergroup level. The column ‘No’ refers to the number of roles the overlapping species held, i.e., the number of bipartite networks (or interaction types in the same network) the species occurred in. The column ‘Present as’ displays which interaction types, or networks the overlapping species occurred in (i.e., what roles they held). Only taxa with full species name determined were included to avoid overestimating overlapping species (e.g., *Amoebophrya* sp. and similar were excluded). Abbrevations: host of par = host of parasite, host of symb = Host of symbiont.

### The SAR supergroup: dominance in literature vs. dominance in environmental studies

The SAR supergroup heavily dominated PIDA and we examined it in depth. Within the SAR supergroup the well-known and species rich Diatomea (Stramenopiles), Dinoflagellata, and Ciliophora (both Alveolata) are prevalent (Fig. 5). Since SAR is also known to dominate environmental sequencing studies [45–47], we compared the SAR records in PIDA with one of the most well-known recent environmental diversity studies; the *Tara Oceans* survey [45]. We identified taxonomic groups that displayed high diversity and abundance in the *Tara Oceans* survey, but that had few entries in PIDA (yellow circles in Fig. 5). Within Stramenopiles, the groups that appeared to be especially underrepresented compared with environmental sequencing data were the Labyrinthulomycetes and Marine Stramenopiles (MASTs). These groups were represented by ~1100 Operational Taxonomic Units (OTUs, a species proxy) in the environmental study by de Vargas et al. [35], while comprising only 12 entries in PIDA (~0.5% of the total entries). The most prominent underrepresentation of alveolates in PIDA compared with *Tara* Oceans was Syndiniales (MALV II). Even though Syndiniales were relatively numerous in PIDA (200 entries; ~8% of the total entries), the MALV II/Syndiniales comprised an astonishing ~5600 OTUs in the study by de Vargas et al. [45]. Within Rhizaria the foraminifers were fairly well represented in PIDA (330 entries, >55 unique species) compared with *Tara Oceans* (~250 OTUs) and also compared with a recent study of Morard et al. [48]. The other rhizarian groups such as Acantharia, Polycystinea, and Cercozoa were, however, poorly represented in PIDA compared with the diversity in the *Tara* Oceans study (~100–150 entries vs. ~1000 to >5000 OTUs in *Tara Oceans*). Apicomplexa did not comprise many entries in PIDA because these parasites only infect multicellular (metazoan) hosts. Therefore, the few apicomplexans that are present in PIDA are recorded as *hosts* of parasites, symbionts, or as prey.

## Discussion

As expected, the data assembled in PIDA reflected biases related to ease of study (e.g., accessibility to the interaction, cell size, organismal abundance), research interests and historical traditions, as well as available technology. Yet, PIDA represents a useful framework to investigate protist ecological interactions that should be expanded and improved with future studies and observations. In particular, future interconnections between PIDA and molecular studies should enhance the power of both and lead to more solid conclusions. Despite its biases, PIDA can be used to initialize biological hypotheses that should be further tested, and can serve as a guide for future experiments on interactions in underrepresented protists groups.

Our comprehensive bibliographic survey shows that microbial interactions are spread across all major eukaryotic groups as well as across the main bacterial (e.g., Cyanobacteria, Bacteroidetes, Proteobacteria, and Firmicutes) and archaeal (Halobacteria, Methanobacteria, and Methanomicrobia) lineages. All major protistan groups were involved in interactions in one or multiple ways; as hosts, symbionts, parasites, predators, and/or prey (Fig. 1a–d). The well-known representatives of the SAR supergroup (i.e., Stramenopiles, Alveolata, and Rhizaria) were however overrepresented compared with the other ‘supergroups’ in PIDA. Within SAR the distribution was further skewed toward certain well-characterized and species-rich lineages, such as dinoflagellates, ciliates, and diatoms. SAR members have historically gained much attention since they hold important roles as primary producers, parasites, symbionts, and predators/grazers [49]). In particular, the dinoflagellate and diatom lineages include many species that hold key roles as primary producers, fixating substantial amounts of atmospheric CO_2_ in the surface ocean [50], and have consequently been the subject of many studies. Dinoflagellates also include species that can form harmful algal blooms (HABs) [51], producing toxins that can have devastating effects on fisheries and aquaculture, making the research on their ecology and life cycles a priority [52]. Other heterotrophic dinoflagellates and ciliates are among the most important microzooplankton predators in the ocean, consuming between 60 and 80% of the primary production every day at a global scale [53–56]. Although the SAR supergroup dominated PIDA, our comparison of the SAR records with the *Tara* Oceans data [45] revealed that there are several SAR lineages, which are abundant and diverse in the marine realm, that were underrepresented when it comes to characterization of their ecological roles as interactors in aquatic environments, such as Labyrinthulomycetes and MAST (Stramenopiles), Syndiniales (MALV II; Alveolata), Acantharia, Polycystinea, and Cercozoa (Rhizaria).

Compared with environmental studies there were also several other protist lineages that were underrepresented in PIDA. Fungi, for example, have been shown to be diverse in environmental HTS surveys of several aquatic environments [57–59] and some of these have been proposed as important parasites of protists [60, 61]. Judging from the scant number of entries in PIDA and the relatively broad host ranges that these organisms had, we suggest that future investigations should focus on revealing more about the ecological function of aquatic fungi. Another underrepresented lineage in PIDA is Excavata. In the *Tara* Oceans surveys the diplonemids were shown to be hyperdiverse, with more than 12 000 OTUs, while they have only four entries in PIDA. It was not surprising that diplonemids were poorly represented in PIDA since their immense diversity was only recently discovered [45], and because little is known about the lifestyle of these excavates [62]. But it underlines that diplonemids and other excavates represent a black box also when it comes to ecological interactions.

Protist predation or grazing is crucial for channeling carbon and energy to higher trophic levels [55, 63] as well as for the release of dissolved nutrients to the base of aquatic food webs [64]. The bipartite network analysis of the predator–prey interactions in PIDA indicated that predation as an ecological strategy is not directed toward either specialization nor generalization, but that predators are ‘multivorous’ and feed on several different prey organisms. Instead of hunting for prey many organisms depend on other strategies for resource acquisition that involve more intimate relationships, such as the interaction between parasites or symbionts and their hosts. These intimate interactions have evolved independently from freeliving ancestors multiple times in diverse and evolutionary unrelated protist lineages. To develop such intimate interactions requires a high degree of specialization as it necessitates a metabolic dialog between the interacting organisms. Our bipartite network analyses for symbiont–host and parasite–host interactions showed that many of the symbiont and parasite species seemed to be moderately specialized.

The importance and scientific relevance of symbiosis was reflected by the great variety of symbiotic interaction we found. Several protists harbor microbial symbionts (eukaryotic and prokaryotic) that provide e.g., carbohydrates (through photosynthesis), vitamins, Nitrogen (through N_2_-fixation), and defense to their hosts, in exchange for other nutrients, vitamins, and protection [1, 4]. An example of this is Cyanobacteria that live inside their protist hosts (e.g., diatoms, dinoflagellates, or radiolarians) and provide photosynthesis products [65, 66] or nitrogen through nitrogen fixation [67–69], in exchange for protection and/or nutrients. There were also many records of heterotrophic bacteria engaged in symbiosis with protists in PIDA, where bacteria provide their hosts with vitamins and other types of nutrients in exchange for photosynthesis products (carbohydrates), nutrients, or protection. Such types of symbiotic interactions have been demonstrated for several relationships between bacteria and microalgae [70–72]. One example is the relationship between the diatom *Pseudo-nitzschia multiseries* and *Sulfitobacter* sp. SA11. *Pseudo-nitzschia multiseries* secretes organic carbon and a sulphonated metabolite called taurine which is taken up by the *Sulfitobacter* sp., and the *Sulfitobacter* sp. bacteria respond by secreting ammonium for the diatom and then switch their preference from ammonium to nitrate, thereby promoting the growth rate of both partners involved in the symbiosis [73]. There were remarkably few studies demonstrating symbiotic relationships between two or more heterotrophic protists in the aquatic environment. The only records we found all represented the same type of symbiotic relationship between the parasite *Neoparamoeba perurans* and its kinetoplastid endosymbionts. *Neoparamoeba perurans* is a well-studied organism since it causes disease in salmon, and consequently is a threat for aquaculture [74–77].

Compared with the other three categories (symbiont–host, parasite–host, and predator–prey) the unresolved interactions were heavily dominated by bacteria–protist interactions, mainly between Alpha- or Gammaproteobacteria interacting with Ciliophora, Dinoflagellata (both Alveolates), or Diatomea (Stramenopiles). Since the functional role of the relationship between these bacteria and protist hosts is not determined, future studies should focus on elucidating these interactions as they could be important components in protist holobiomes. The holobiome concept includes the host and all associated microbes (eukaryotes, prokaryotes, and viruses). There has been a growing awareness that microbes associated with larger hosts (e.g., humans) have profound effects on the host’s health and development [22, 78], and this most likely applies to protist holobiomes too.

Parasites are present in most phylogenetic groups and hold important roles in ecosystems where they can for instance alter both the structure and dynamics of food webs [79, 80]. Parasites are likely largely underrepresented in studies of microbial interactions with only 18% of the entries in PIDA. This is especially prominent in the light of recent results from environmental DNA surveys, which indicate that parasites are particularly diverse and abundant in marine as well as terrestrial ecosystems [9, 45, 81, 82]. Cercozoan parasites were only described from diatoms, although several of their close relatives are known to parasitize a plethora of macroscopic hosts [83–86]. Likewise, parasites belonging to different stramenopile lineages such as Peronosporomycetes (oomycetes), Labyrinthulomycetes, and Pirsonia were also registered to mostly infect diatoms (Fig. 1d). This probably reflects that diatoms have been the subject of more scientific studies than other protist hosts, although the true diversity of parasites infecting diatoms is likely larger than what is currently known [84].

The bipartite network analyses indicated that there were some parasites in PIDA that infected many different hosts (i.e., broad host range). But in general, the majority of parasites in PIDA appeared to infect few hosts, and altogether, parasite–host interactions seemed to be slightly more dominated by specialized interactions than in symbiont–host networks. For symbionts and parasites, the observed patterns could indicate that several studies have investigated these relationships from ‘the parasite/symbiont point of view’, and consequently, well-known taxa (e.g., the parasite *Amoebophrya*) have been investigated more thoroughly leading to broader host ranges. In contrast, several other symbionts/parasites have been detected only associated with one host, pointing to many specialized ‘one-to-one’ relationships in the microbial world. It could also be speculated that this pattern results from accidental detections of parasites or symbionts. For instance, research on diatoms would from time to time observe cells that are infected with a parasite or that host a symbiont, without looking more into the host range of these parasites or symbionts, as that was not the original focus of the study. Both the *symbiont*–*host*, *interactor*–*host,* and *parasite*–*host* categories in PIDA are dominated by host entries (~double number of hosts compared with the number of interacting partners), and this observation could support that several of the detections of symbionts, parasites, and interactors may represent ‘accidental detections’. The search for a parasite or symbiont’s host range has until recently been like searching for a needle in a haystack.

In conclusion, summarizing the data on ecological interactions involving aquatic protists and other microbes from the past ~150 years allowed us to obtain a unique overview of the known interactions and derive relevant biological hypotheses. Despite the biases and knowledge gaps we identified, PIDA can be used for multiple purposes in future studies (which is beyond the scope of this work), for example: (1) to identify the functional role of a microbe using taxonomically annotated environmental DNA sequences, (2) to investigate whether ecological interaction hypotheses that derive from association networks [23] are supported by previous studies in PIDA, and (3) to obtain information about the host range of a particular parasite, the predators of a specific prey, or the symbionts from a given host. Last but not least, our work identifies knowledge gaps that could be the focus of future research.

## Supplementary information

Supplementary material
