# Supplementary material for: The planktonic protist interactome: where do we stand after a century of research?
Source: ISME J. 2019 Nov 4;14(2):544–59. doi: 10.1038/s41396-019-0542-5 (PMC6976576; doi:10.1038/s41396-019-0542-5)
Supplement: Supplementary file 1 — Supplementary material [file 41396_2019_542_MOESM1_ESM.pdf]

Supplementary information

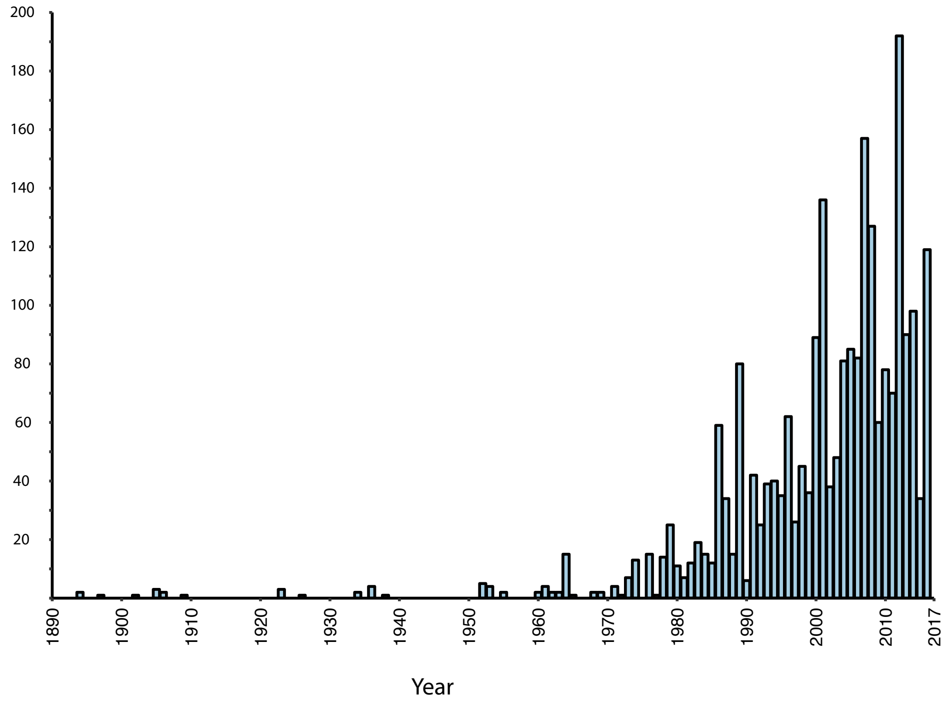

Supplementary figure 1: Number of scientific publications per year included in PIDA.

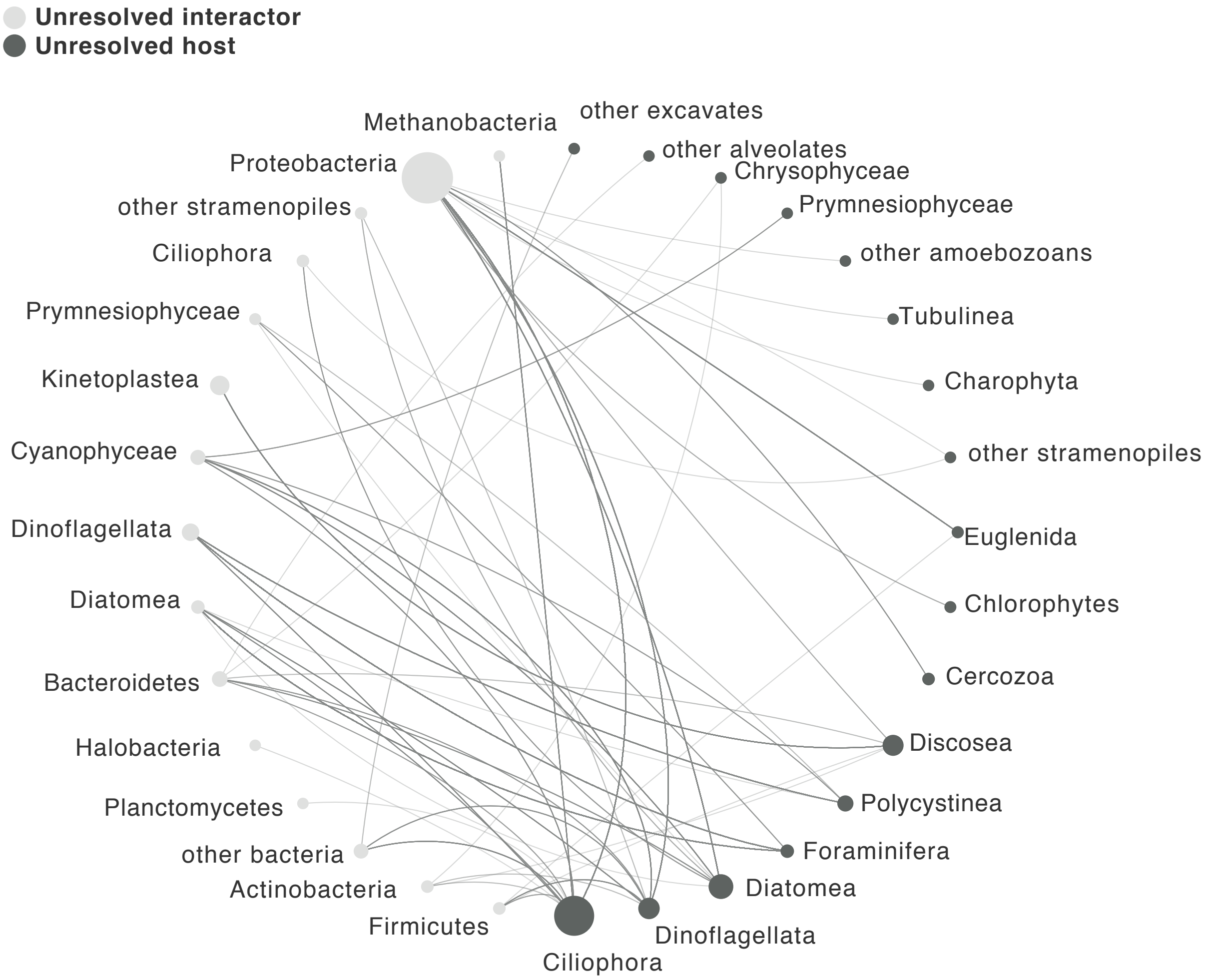

Supplementary figure 2. Overview of the Unresolved interactions in PIDA. Network based on the 338 unresolved interaction entries in PIDA. Unresolved interactor organisms are shown in dark grey and the unresolved host organisms are shown in light grey. Sizes of nodes and number of edges (i.e. lines) represent number of interactions between unresolved interactor and host taxa.

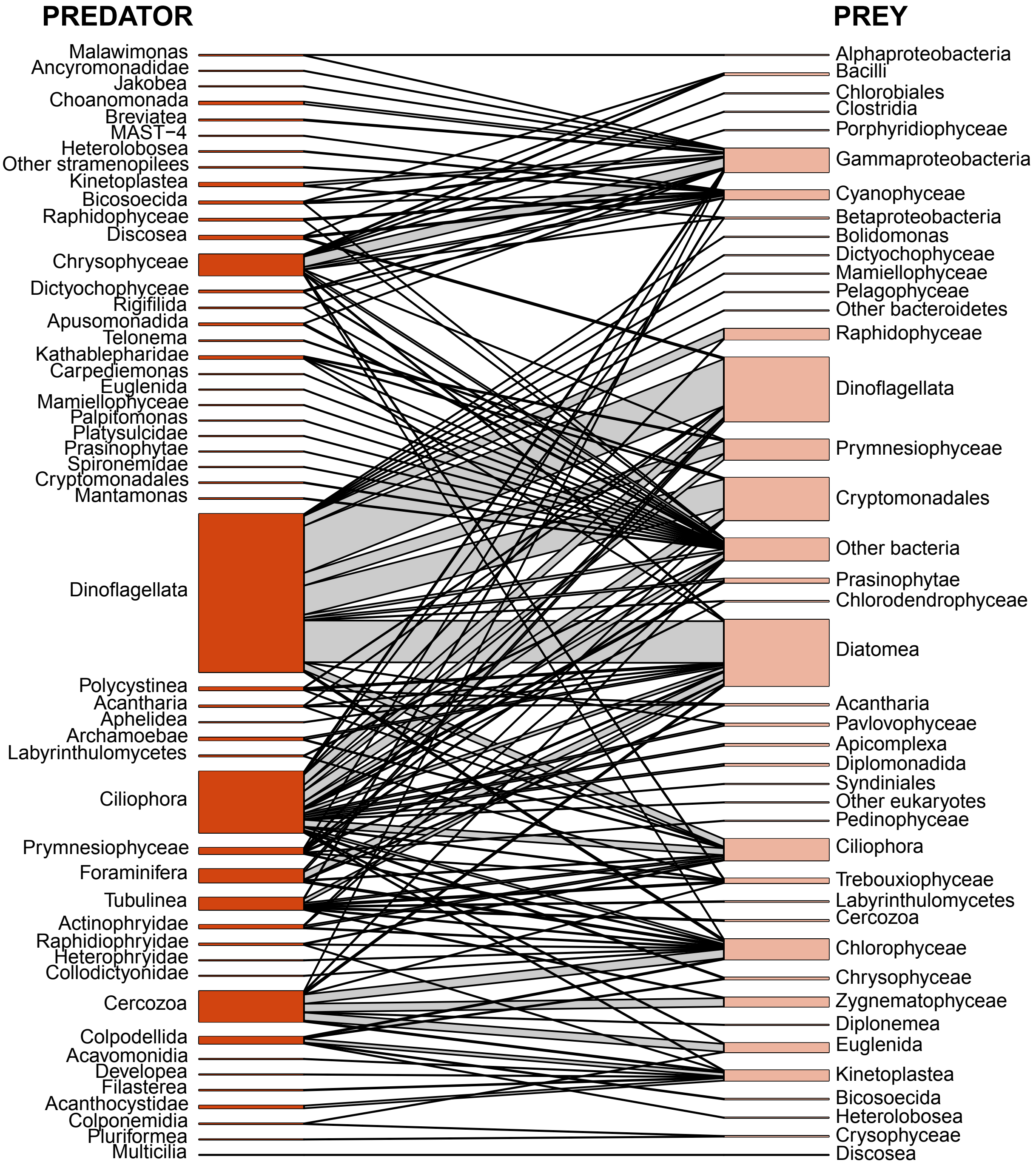

Supplementary figure 3. Bipartite network of Predator-Prey interactions in PIDA, at the 'phylum level' (corresponding to the third taxonomic level in PIDA). Predator organisms are to the left (dark orange) and prey organisms are to the right (light orange). Sizes of nodes and number of edges (i.e. lines) represent number of interactions between prey and predator taxa.

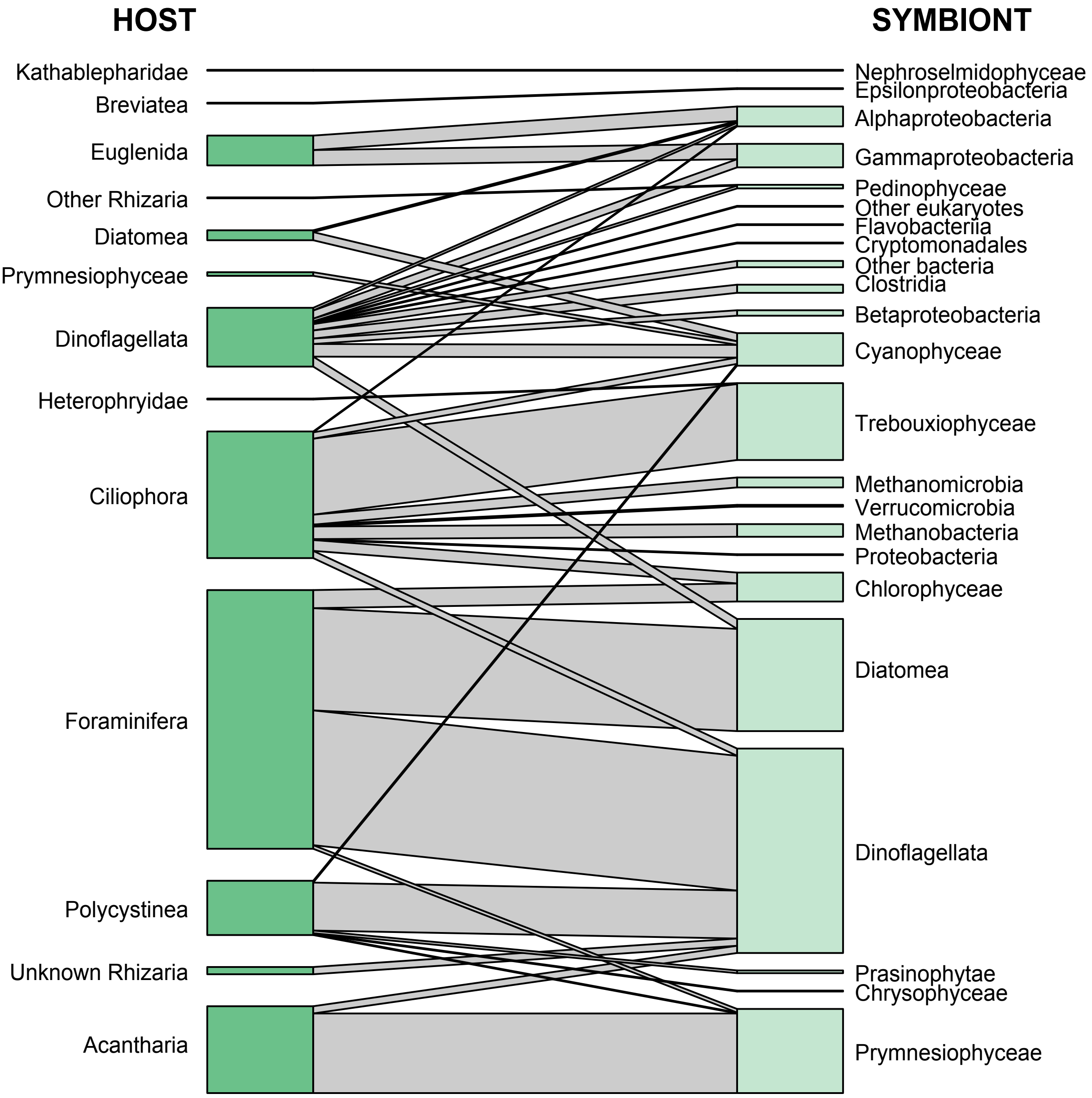

Supplementary figure 4. Bipartite network of Symbiont-Host interactions in PIDA, at the 'phylum level' (corresponding to the third taxonomic level in PIDA). Host organisms are to the left (dark green) and symbiont organisms are to the right (light green). Sizes of nodes and number of edges (i.e. lines) represent number of interactions between symbiont and host taxa.

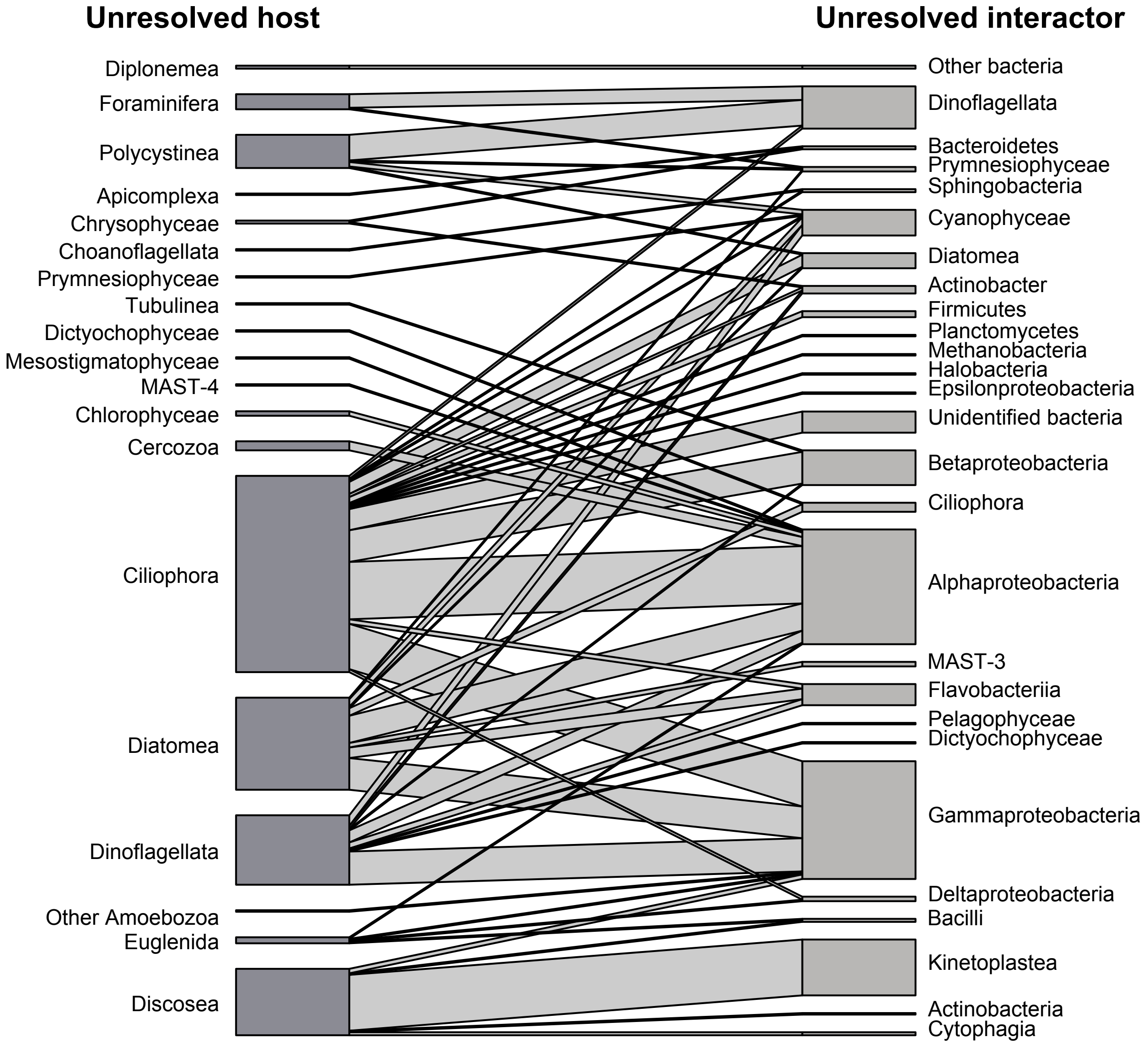

Supplementary figure 5. Bipartite network of Unresolved Interactor-Host interactions in PIDA, at the 'phylum level' (corresponding to the third taxonomic level in PIDA). Unresolved host organisms are to the left (dark grey) and unresolved interactor organisms are to the right (light grey). Sizes of nodes and number of edges (i.e. lines) represent number of interactions between unresolved interactor and host taxa.

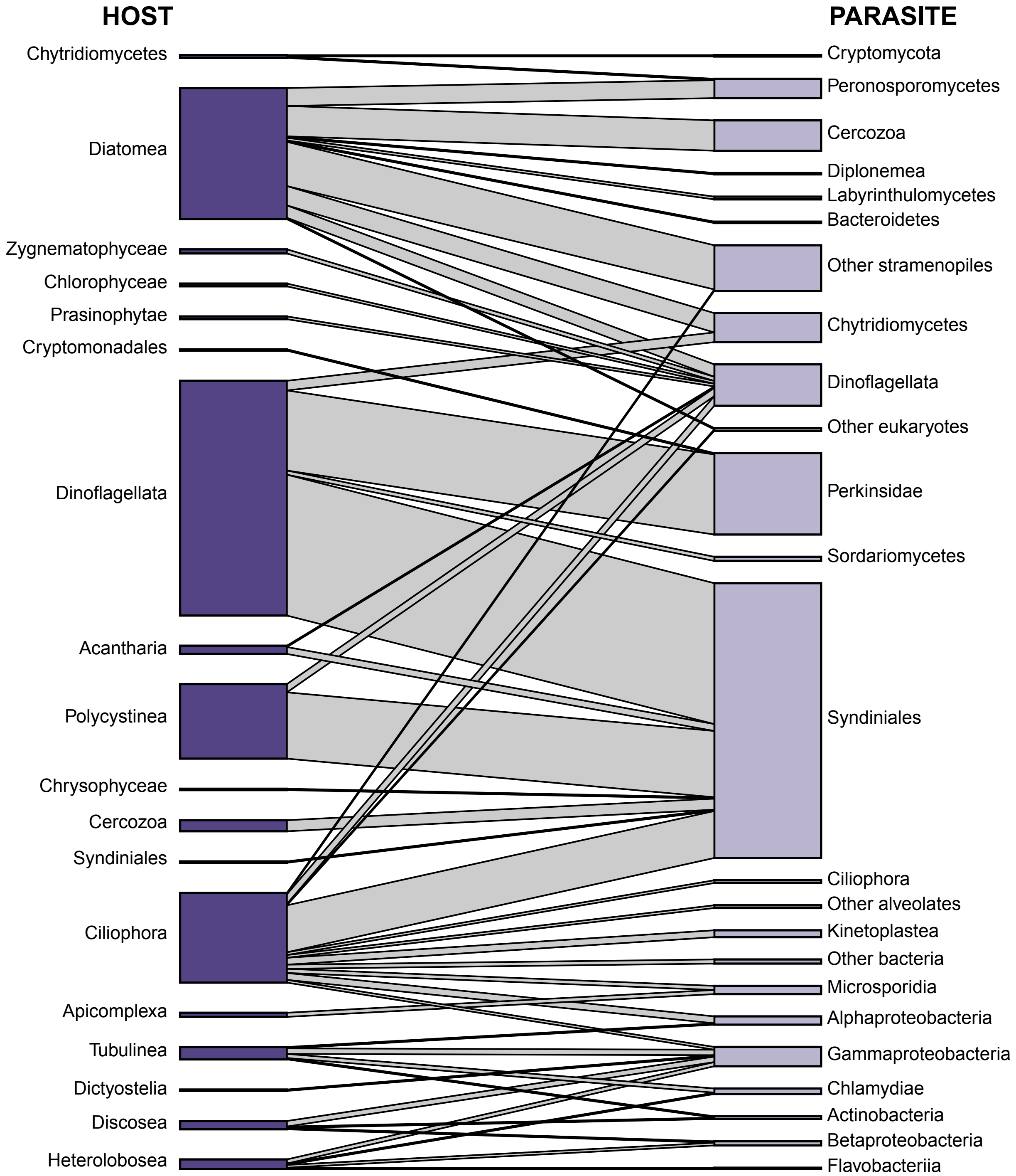

Supplementary figure 6. Bipartite network of Parasite-Host interactions in PIDA, at the 'phylum level' (corresponding to the third taxonomic level in PIDA). Host organisms are to the left (dark purple) and parasite organisms are to the right (light purple). Sizes of nodes and number of edges (i.e. lines) represent number of interactions between parasite and host taxa.

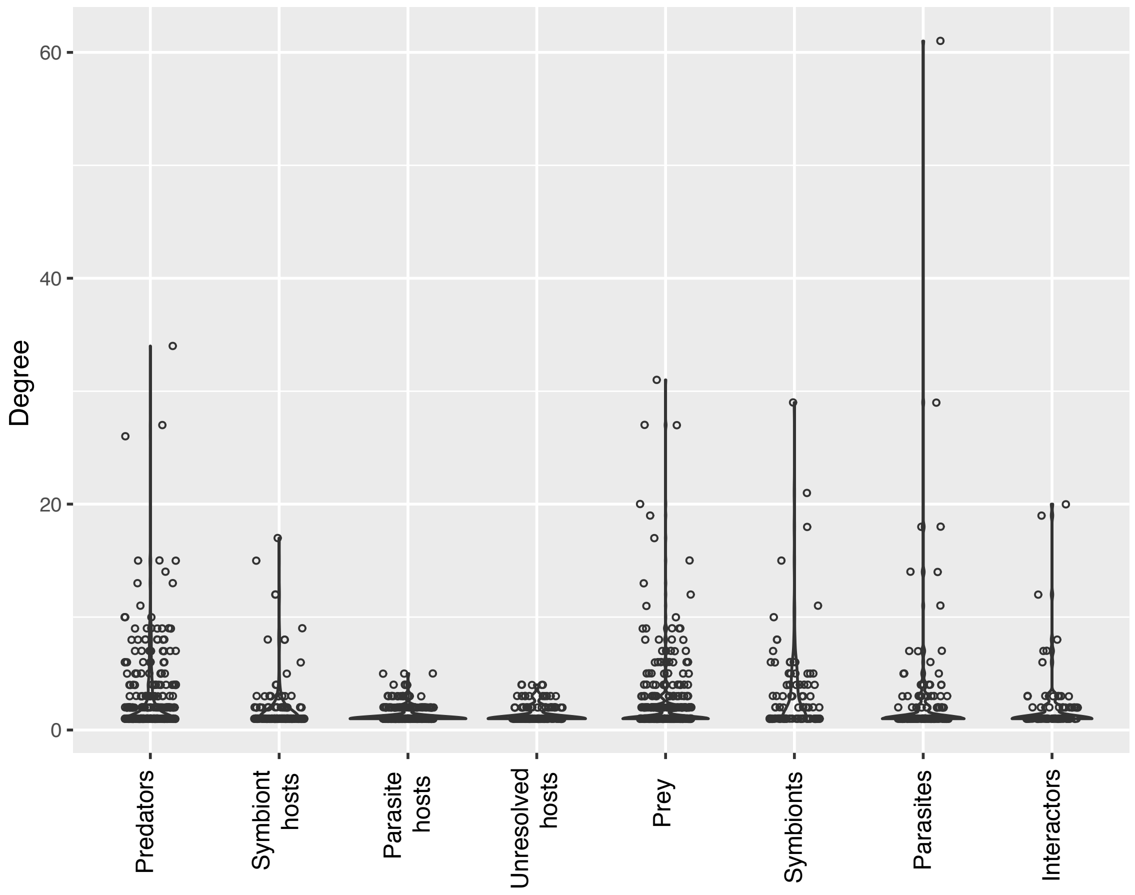

Supplementary figure 7. Degree displaying the number of links/edges that each species has with partners in the other level of the bipartite networks, e.g. the parasites to the far right (light purple colour) are represented with some species with high degree, i.e. registered to parasitize many different host species.
